# BOLD fMRI and hemodynamic responses to somatosensory stimulation in anesthetized mice: spontaneous breathing vs. mechanical ventilation

**DOI:** 10.1002/nbm.4311

**Published:** 2020-04-15

**Authors:** Hyun‐Ji Shim, Joonyeol Lee, Seong‐Gi Kim

**Affiliations:** ^1^ Center for Neuroscience Imaging Research (CNIR) Institute for Basic Science (IBS) Suwon 16419 Republic of Korea; ^2^ Department of Health Sciences and Technology, SAIHST Sungkyunkwan University Seoul 06355 Republic of Korea; ^3^ Department of Biomedical Engineering Sungkyunkwan University Suwon 16419 Republic of Korea

**Keywords:** anesthesia, BOLD fMRI, cerebral blood volume, forepaw stimulation, isoflurane, ketamine, vascular reactivity

## Abstract

Mouse functional MRI (fMRI) has been of great interest due to the abundance of transgenic models. Due to a mouse's small size, spontaneous breathing has often been used. Because the vascular physiology affecting fMRI might not be controlled normally, its effects on functional responses were investigated with optical intrinsic signal (OIS) imaging and 9.4 T BOLD fMRI. Three conditions were tested in C57BL/6 mice: spontaneous breathing under ketamine and xylazine anesthesia (KX), mechanical ventilation under KX, and mechanical ventilation under isoflurane. Spontaneous breathing under KX induced an average pCO_2_ of 83 mmHg, whereas a mechanical ventilation condition achieved a pCO_2_ of 37‐41 mmHg within a physiological range. The baseline diameter of arterial and venous vessels was only 7%‐9% larger with spontaneous breathing than with mechanical ventilation under KX, but it was much smaller than that in normocapnic isoflurane‐anesthetized mice. Three major functional studies were performed. First, CBV‐weighted OIS and arterial dilations to 4‐second forepaw stimulation were rapid and larger at normocapnia than hypercapnia under KX, but very small under isoflurane. Second, CBV‐weighted OIS and arterial dilations by vasodilator acetazolamide were measured for investigating vascular reactivity and were larger in the normocapnic condition than in the hypercapnic condition under KX. Third, evoked OIS and BOLD fMRI responses in the contralateral mouse somatosensory cortex to 20‐second forepaw stimulation were faster and larger in the mechanical ventilation than spontaneous breathing. BOLD fMRI peaked at the end of the 20‐second stimulation under hypercapnic spontaneous breathing, and at ~9 seconds under mechanical ventilation. The peak amplitude of BOLD fMRI was 2.2% at hypercapnia and ~3.4% at normocapnia. Overall, spontaneous breathing induces sluggish reduced hemodynamic and fMRI responses, but it is still viable for KX anesthesia due to its simplicity, noninvasiveness, and well‐localized BOLD activity in the somatosensory cortex.

## Graphical abstract

Baseline and evoked responses in optical imaging and BOLD fMRI were characterized in mouse somatosensory cortexes under ketamine/xylazine anesthesia. With spontaneous breathing, arterial dilation in response to forepaw stimulation was much larger and faster than venous dilation. Mechanical ventilation allowed us to maintain the mice in normal physiological conditions, whereas spontaneous breathing induced severe hypercapnia. Mechanical ventilation provided larger and faster functional responses, but spontaneous breathing is still viable for ketamine/xylazine‐anesthetized mouse fMRI studies due to its simplicity.

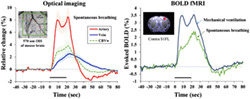

**Abbreviations used**
CBF: cerebral blood flow
CBV: cerebral blood volume
CBVw: CBV‐weighted
EPI: echo planar imaging
GLM: general linear model
HR: heart rate
Iso: isoflurane
KX: ketamine and xylazine
MABP: mean arterial blood pressure
MV: mechanical ventilation
OIS: optical intrinsic signal
PCO_2_: partial arterial pressure of CO_2_; PO_2_, partial arterial pressure of O_2_
*R*: reflectance image intensity
ROI: region of interest
S1FL: primary somatosensory forelimb
SaO_2_: arterial oxygen saturation level
SB: spontaneous breathing

## 1 INTRODUCTION

Blood oxygenation level‐dependent (BOLD) functional MRI (fMRI) in rodents has been used to understand the biophysics and neural sources of fMRI signals and to investigate brain functions. BOLD fMRI of anesthetized mice has been of particular interest due to the abundance of transgenic models, easy manipulation of genomes, and cost efficiency, but the literature findings are inconsistent.^1^, ^2^, ^3^, ^4^, ^5^, ^6^, ^7^ Because fMRI measures relative changes in hemodynamics, it is highly affected by the baseline condition, which is sensitive to anesthetics, drugs, hormones and physiological factors.^8^, ^9^, ^10^ Therefore, most anesthetized rat fMRI studies determine the baseline physiological conditions using blood pressure and gas analyses and control them using mechanical ventilation with intubation. When a mean arterial blood pressure level ranges about between 50 and 160 mmHg, cerebral blood flow (CBF) is regulated at a constant level.^11^, ^12^, ^13^, ^14^ Since CBF is highly related to arterial CO_2_ pressure (pCO_2_), pCO_2_ is maintained at 30‐45 mmHg.^15^, ^16^, ^17^ As a result, fMRI studies carried out in various laboratories can produce consistent results.^18^, ^19^, ^20^, ^21^, ^22^

In mouse fMRI, cardiac and respiratory rates are generally measured along with the arterial oxygen saturation level, but blood pressure and gases are seldom detected due to the difficulty of arterial catherization and repeated blood sampling in a mouse's small body.^6^, ^23^, ^24^, ^25^, ^26^ Therefore, vascular physiology cannot be controlled precisely, which might explain the variability in mouse fMRI findings. We have successfully obtained localized functional activity in response to forepaw stimulation in ketamine and xylazine‐anesthetized mice, but its time‐to‐peak was around the offset time of the 20‐second stimulation period, which is much longer than that in rat forepaw stimulation data.^18^, ^19^, ^20^, ^23^, ^24^ In many mouse fMRI studies^1^, ^3^, ^25^, ^27^ including ours with ketamine and xylazine anesthesia,^23^, ^24^ only heart rate (HR) and breathing patterns were measured under spontaneous breathing with a supplementary oxygen supply, which decreases pH and increases pCO_2_ considerably.^28^ When the pCO_2_ level increases, the baseline CBF also increases by dilating vessels, inducing reduced magnitude and sluggish dynamics in the hemodynamic responses.^29^, ^30^, ^31^ Therefore, it is important to determine baseline conditions and their effects on hemodynamic responses.

To evaluate the effects of baseline conditions on evoked responses, we evaluated three different conditions: (1) spontaneous breathing under ketamine/xylazine, (2) mechanical ventilation under ketamine/xylazine, and (3) mechanical ventilation under commonly used isoflurane. First, to determine baseline physiological conditions, we measured arterial pCO_2_, oxygen partial pressure (pO_2_) and pH, and then we compared the baseline arterial and venous diameters between the three conditions. Spontaneous breathing induces severe acidosis and hypercapnia, while mechanical ventilation controls normal vascular physiology. Second, functional cerebral blood volume (CBV)‐weighted optical imaging to 4‐second forepaw stimulation, was obtained in the three conditions, and the dynamics and magnitude changes were compared. Arterial and venous vessel dilations were also compared. Since small functional responses were observed under isoflurane, only two conditions with ketamine/xylazine were further used for determining vascular reactivity and functional responses to 20‐second forepaw stimulation. Third, since hypercapnia reduces vascular responses, the vascular reactivity to the vasodilator acetazolamide was compared in the two breathing conditions of ketamine/xylazine‐anesthetized mice. Fourth, functional CBV‐weighted optical imaging, and arterial and venous vessel responses to forepaw stimulation were obtained in the two breathing conditions, and the dynamics and magnitude changes were compared. Fifth, BOLD fMRI responses to forepaw stimulation at 9.4 T were determined under the two breathing conditions under ketamine/xylazine anesthesia. Well‐localized BOLD activity was detected in the contralateral somatosensory cortex. Faster and larger BOLD responses were observed in the normocapnic mechanical ventilation condition than in the hypercapnic spontaneous breathing condition.

## 2 MATERIALS AND METHODS

### 2.1 Animal preparation and surgery

#### 2.1.1 Animals

All animal experiments were conducted with approval by the Institutional Animal Care and Use Committee of Sungkyunkwan University in accordance with the standards for humane animal care and use as set by the Animal Welfare Act and the National Institutes of Health Guide for the Care and Use of Laboratory Animals. A total of 69 adult male C57BL/6 mice (23‐30 g, aged 2‐4 months; Orient Bio, Korea) were used; two mice in the fMRI groups were excluded, because each animal's condition was not properly maintained due to a heating pad malfunction. In the isoflurane experimental condition, animals were initially induced by 4% isoflurane (Hana, Korea), prepared with 2%‐2.5% isoflurane during surgery and positioning on a cradle, then maintained at 1.0%‐1.1% during the experiments. For the ketamine and xylazine condition, an intraperitoneal (IP) injection of 100 mg/kg of ketamine (Yuhan, Korea) and 10 mg/kg of xylazine (Rompun, Bayer, Korea) was used for the initial induction, and a subsequent IP bolus injection of 25 mg/kg of ketamine and 1.25 mg/kg of xylazine (KX) was given about every 45 minutes as needed, based on physiological signal changes previously described in detail.^24^

#### 2.1.2 Surgical preparation

1. Craniotomy. For intrinsic optical imaging, a craniotomy was performed over the left or right somatosensory area under ketamine and xylazine anesthesia (100 and 10 mg/kg, respectively, IP), according to previously reported methods.^24^ Briefly, a small amount of lidocaine (1 mg/kg, lidocaine HCl 2%, Daihan, Korea) was administered subcutaneously to the head to relieve pain before scalp dissection. A section ~4 mm in diameter centered about 2 mm lateral and 0.1 mm rostral from the bregma was removed using a dental drill, and then the hole was covered with a cover glass (Deckglaser, Marlenfeld, Lauda‐Konigshofen, Germany). The rim of the cover slip was secured by applying dental resin (OA2, DENTKIST, Gunpo, Korea). After the surgery, Metacam (5 mg/kg, meloxicam, Boehringer Ingelheim, Korea) was injected subcutaneously to relieve inflammation and pain, and then the animals were allowed to recover from anesthesia and were returned to their cages. Experiments were performed >3 weeks after the craniotomy surgery.
2. Intubation and catheterization. For the mechanical ventilation studies, intubation was performed after the administration of general anesthesia (isoflurane or ketamine/xylazine mixture). A 22‐gauge intravascular catheter (KOVAX‐CATH, Korea Vaccine, Seoul, Korea) was inserted into the trachea via guidance with a surgical light and secured to the mouth with a suture. Although the animals could recover after the removal of the tracheal catheter, the intubated mice did not gain weight properly within a week, and consequently they were not used for additional experiments. Arterial catheterization was performed to obtain a blood gas analysis and blood pressure. Tubes of three different sizes (polyurethane BTPU014, polyethylene PE10 and PE50 tubing) were connected and inserted into the left femoral artery. To measure vascular reactivity to a vasodilator in six mice, a tail vein was catheterized with a 30G needle connected to PE10 tubing. To deliver the ketamine and xylazine mixture intermittently, a 30G needle connected to a PE10 tube was placed intraperitoneally.

#### 2.1.3 Ventilator settings

Oxygen and air (15%:85%) were supplied through a ventilator (SAR‐1000, CWE, Ardmore, OK for experiments in all mechanical ventilation conditions and spontaneous breathing condition experiments in optical intrinsic signal [OIS] imaging, or TOPO, Kent Scientific, Torrington, CT for the fMRI studies with spontaneous breathing). For spontaneous breathing, gases were supplied through a tube or nose cone with a mask (OIS imaging and fMRI, respectively) at 1 L/minute. In the mechanical ventilation case, exactly the same tubing length (4.45 m, ID: 1.6 mm) was used for both the OIS and fMRI studies. To identify the proper ventilator settings for maintaining normal physiological conditions, extensive preliminary studies were performed with blood gas analysis (i‐STAT Portable Clinical Analyzer; Abbott Point of Care, Princeton, NJ). The determined ventilator settings were an inspiration/expiration ratio of 35%:65%, a respiratory rate of 160 (ketamine/xylazine) or 90 (isoflurane) breaths per minute to match with spontaneous breathing rate. A tidal volume was set to obtain normal blood gas values; 0.0103 × body weight (g) + 0.4117 (i.e., 0.62‐0.7 ml) for ketamine/xylazine, or 0.0366 × body weight (g) + 0.1095 (i.e., 0.91‐1.14 ml) for isoflurane.

#### 2.1.4 Stimulation

A pair of needle (30G) electrodes was placed in the right or left forepaw between digits 2 and 4 for electrical stimulation. Electrical pulse stimuli were given with a constant current stimulation isolator (ISO‐Flex, AMPI, Jerusalem, Israel) triggered by a pulse generator (Master9, AMPI, Jerusalem, Israel). The forepaw stimulation parameters, which were optimized previously,^24^ were a frequency of 4 Hz, a pulse width of 0.5 ms, and current intensity of 0.5 mA. For vascular stimulation without neural activity, a 30 mg/kg bolus of acetazolamide (Zoladin, BC World Pharm. Co, Korea) was injected into the tail vein.

### 2.2 Experimental designs

Under ketamine and xylazine anesthesia, spontaneous breathing (KX‐SB) and mechanical ventilation (KX‐MV) were used to evaluate the effects of the breathing methods on vascular physiology and functional studies. Experiments with isoflurane anesthesia under mechanical ventilation (Iso‐MV), commonly used in rodent functional studies, were also performed for comparisons with the KX anesthesia conditions. During the functional studies, electrocardiogram and motion‐sensitive respiration signals were monitored continuously (Model 1030, Small Animal Instruments, Stony Brook, NY for fMRI and PhysioSuite, Kent Scientific for OIS) and recorded using a data acquisition system (Acknowledge, Biopac Systems, Goleta, CA). Throughout the experiments, each animal's body temperature was maintained at 37 ± 0.5°C. The following experiments were performed.

1. Evaluation of baseline physiological conditions

Basal animal conditions were measured in three separate animal groups (total *n* = 24). Through the femoral artery line, arterial blood pressure was measured and recorded. An arterial blood volume of 0.1 ml was withdrawn twice at a 1‐hour interval, and arterial blood gases (pO_2_, pCO_2_ and pH) were measured with a portable blood gas analyzer (i‐STAT Portable Clinical Analyzer). The mean arterial blood pressure and cardiac pulsation rate were averaged from the 1‐hour arterial blood pressure data, excluding values exceeding two standard deviations from the mean, and the two blood gas measurements were averaged. Arterial oxygen saturation levels (SaO_2_) were determined from pO_2_ with an oxyhemoglobin dissociation curve of mouse.^32^

- Baseline vessel diameter and evoked CBV‐weighted OIS

Intrinsic optical imaging was performed to compare vessel diameters and CBV‐weighted (CBVw) OIS responses induced by forepaw stimulation or acetazolamide. Three experiments were performed: baseline and evoked optical response to 4‐second forepaw stimulation, evoked optical response to 20‐second forepaw stimulation, and vascular reactivity. Multiple experiments were performed in some animals: (i) baseline and evoked optical response to 4‐second stimulation were obtained under KX‐SB, KX‐MV and Iso‐MV conditions. To compare responses in the same animals, the KX‐SB studies were performed first in 12 animals, then 1 week later, mechanical ventilation experiments were carried out under ketamine/xylazine or isoflurane anesthesia. Note that the other order was not used due to the long‐lasting detrimental effect of intubation. Each stimulus trial consisted of a 5‐second prestimulus, 4‐second forepaw stimulus, and a 51‐second poststimulus period that was repeated 15 times; (ii) because BOLD fMRI studies used long stimulation duration due to a limited temporal resolution, 20‐second forepaw stimulation was also used in the two KX conditions (*n* = 6 in the KX‐SB condition used for 4‐second stimulation, and *n* = 5 for KX‐MV) for comparison with BOLD fMRI data. Each of those trials consisted of a 40‐second prestimulus, 20‐second stimulus and a 60‐second poststimulus period, and they were repeated 10 times; (iii) to compare vascular reactivity under the hypercapnic KX‐SB condition (*n* = 6) and normocapnic KX‐MV condition (*n* = 5), OIS images were obtained during 5 minutes before and 10 minutes after the intravenous bolus injection of the 15 mg/kg vasodilator acetazolamide, a potent inhibitor of carbonic anhydrase.

- Evoked BOLD fMRI responses under ketamine and xylazine anesthesia

To investigate breathing condition‐dependent BOLD responses (spontaneous breathing vs. mechanical ventilation), BOLD fMRI was performed in separate animals under a ketamine and xylazine mixture. In our previous fMRI studies of mechanically ventilated isoflurane‐anesthetized mice,^24^ we did not observe reliable localized activity with forepaw stimulation; thus we used only ketamine‐xylazine anesthesia for the activation studies. Each stimulus trial consisted of a 40‐second prestimulus, 20‐second stimulus and a 60‐second poststimulus period, and trials were repeated 13‐18 times. In the KX‐MV case, an arterial blood gas analysis was performed once before the fMRI experiment to confirm normal physiological conditions.

### 2.3 Data acquisition

#### 2.3.1 CBV‐weighted OIS imaging

OIS images were obtained using Imager 3001 data acquisition software (Optical Imaging, Israel) with an MVX‐10 microscope (Olympus, Tokyo, Japan) and a 572 ± 15 nm wavelength filter, following previously reported OIS methods.^24^ The transmission light was filtered and delivered to the exposed somatosensory cortex by light guides connected to an LED light source (CLS150, Leica, Korea). This wavelength is an isosbestic point for hemoglobin, making the OIS data sensitive to changes in the total amount of hemoglobin and therefore representative of CBV. OIS data with 660 × 612 (600 × 612 for drug studies) pixels were acquired with a field of view of 1.3 × 1.2 mm^2^ (1.2 × 1.2) at 10 frames per second (fps) for 4‐second stimulation experiments, 2 fps for 20‐second stimulation experiments, and 1 fps for vascular reactivity experiments. The frames were stored as 300 × 306 pixel (200 × 204) images after binning by 2 × 2 pixels (3 × 3 for drug studies).

#### 2.3.2 9.4 T BOLD fMRI

All MRI experiments were performed on a horizontal bore 9.4 T/30 cm Bruker BioSpec MR system (Billerica, MA) with an actively shielded 12‐cm diameter gradient insert operating with a maximum strength of 66 Gauss/cm and a rise time of 141 μs according to previously reported fMRI methods.^24^ A quadrature birdcage coil (86 mm inner diameter) was used for excitation, and an actively decoupled Bruker planar surface coil (10 mm inner diameter) positioned on top of the mouse's head was used for detection. The magnetic field homogeneity was globally shimmed with the field map method, and then a local shim was optimized using the MAPSHIM protocol with an ellipsoid shim volume covering the cerebrum (ParaVision 6, Bruker BioSpin).

All fMRI data in the two breathing conditions were acquired using a single‐shot gradient echo (GE) echo planar imaging (EPI) sequence with the following parameters: echo time spacing = 320 μs, sampling frequency = 300 KHz, TR/TE = 1000/16 ms, flip angle = 60°, number of averages = 1, field of view = 18 (readout) × 12 (phase encoding) mm^2^, matrix = 96 × 64, in‐plane resolution = 188 × 188 μm^2^, slice thickness = 500 μm, number of contiguous coronal slices = 9 without gap, and dummy scans = 10. One hundred twenty volumes (120 seconds) were acquired for the BOLD fMRI.

### 2.4 Data processing

#### 2.4.1 Optical imaging data

All the optical imaging data were analyzed using Matlab (Mathworks, Natick, MA) and Fiji (ImageJ). There were two types of data analysis: (i) the diameter of blood vessels and (ii) the CBVw OIS data. For baseline vessel analysis, the individual trials were averaged first with identical experimental conditions, and then quantitative values were obtained. For evoked vessel analysis, individual trials were separately analyzed first, and then time courses and quantitative values were averaged over multiple trials with identical experimental conditions. Time *t* = 0 was defined as the stimulus onset time. Analyses were performed on each animal separately before group averaging.

#### 2.4.2 Vessel diameter measurement

For each animal, arterial and venous vessels were discriminated by their color and movements; the arterial vessel appeared to be bright red with slight movements caused by cardiac pulsations, while the venous vessels were dark red. Three or four arterial and venous vascular segments >15 μm diameter were identified near the reaction site in the primary somatosensory forelimb (S1FL) region. A three pixel‐wide line roughly orthogonal to the vessel direction was selected for each vascular segment based on visual inspection, and its diameter was measured with the Diameter plug‐in of ImageJ.^33^ Basal vessel diameters were calculated within −3 to −1 seconds prior to each 4‐second stimulation period. In the same animals used for two different conditions (KX‐SB vs. KX‐MV and KX‐SB vs. Iso‐MV), baseline diameters in the KX‐MV and Iso‐MV conditions were normalized by the diameters commonly measured at the KX‐SB condition in the same vascular segment. For reducing the errors due to potential mis‐registrations between images obtained at different conditions, two three‐pixel lines adjacent to the selected line were chosen additionally and their diameters were averaged together (ie nine‐pixel width). Dynamic changes in arterial and venous dilation were determined for the evoked OIS imaging to forepaw stimulation and vascular reactivity. Normalized time courses were obtained, and quantitative values were calculated by averaging values in the prestimulation (i.e., −30 to −10 seconds for long forepaw stimulation and −3 to −1 minutes for drug application) and stimulation (i.e., 5 to 20 seconds for long forepaw stimulation and 3 to 5 minutes postinjection) periods.

#### 2.4.3 CBV‐weighted OIS measurement

Analysis of changes in CBVw OIS was used primarily to compare responses to the forepaw stimuli. Optical maps were obtained for each condition in each animal. To determine the forepaw area, normalized OIS activation maps (Δ*R*(*t*)/*R*) were obtained from subtraction images (Δ*R*(*t*) *= R*(*t*) – *R*). The subtraction images (Δ*R*(*t*)) were obtained by subtracting the prestimulus reflectance image *R* from the time‐dependent reflectance images *R*(*t*), where *R* is the average of the −3 to −1 seconds prestimulus images. For a representative animal, the activation maps were generated at 2‐second intervals from 1 to 17 seconds.

To quantitatively compare hemodynamic responses across different groups, a square region of interest (ROI) of 1 × 1 mm^2^ was selected around the activation focus, which was the pixel cluster with the most significant signal changes. The same ROI was used for two repeated experimental conditions in the same animal. OIS time series were obtained by averaging pixel intensities within the ROI, and they were normalized by their prestimulus baseline intensity (−3 to −1 or −30 to −10 seconds). In that way, we obtained a relative reflectance change (Δ*R*(*t*)/*R*) time series. When hemoglobin contents within the pixel increase with an increase in cerebral blood volume, the absorption of light also increases, resulting in a decrease in reflected light intensity. To match with the polarity of CBV responses, the sign of the OIS time series was reversed. Then peak amplitude and time‐to‐peak were calculated for the 4‐second stimulation condition. The time‐to‐peak was defined as the time at which the stimulus‐induced signal change was highest in the range of 1 to 10 seconds after 4‐second stimulus onset.

#### 2.4.4 Evoked fMRI

BOLD fMRI data were processed with the Analysis of Functional NeuroImages (http://afni.nimh.nih.gov/afni) package and Matlab codes. Animal‐wise functional maps were calculated by averaging all trials in each animal individually with preprocessing and standard general linear model (GLM) analyses. In general, preprocessing was done according to previously reported methods.^24^ To generate statistical t‐value maps, a GLM was applied with the boxcar reference function. Activation maps were shown with voxels passing the thresholds of uncorrected *P* < .005 and cluster size >9. Functional maps were overlaid on original EPI images and also coregistered to the Allen mouse brain atlas. From the functional maps, the number of active voxels in the S1FL region was counted with four different thresholds of *P* < .001, *P* < .005, *P* < .01 and *P* < .05.

In each animal, the contralateral S1FL ROI was defined as a 500 μm radius sphere (23 voxels) at the center of mass of the activated area in each animal‐wise functional map. Time courses were obtained from the S1FL ROI and normalized by the average of the baseline (first 35 volumes), and then trial‐averaging within the same imaging session was performed in each animal. Next, the mean amplitudes were calculated as the mean of signal changes within 7 to 11 and 21 to 25 seconds after stimulus onset.

### 2.5 Statistical analysis

All results are presented as the mean ± standard error of the mean (SEM), unless stated otherwise. The statistical significance was tested using analysis of variance (one‐way ANOVA) with the Bonferroni correction for the blood gas data of three groups. Statistical significance was tested with the student's t‐test in comparisons of the two breathing conditions. A *P*‐value < .05 was considered to be significant.

## 3 RESULTS

### 3.1 Evaluation of baseline physiological condition: KX‐SB, KX‐MV and Iso‐MV

Heart rate, blood pressure and blood gases were compared in three groups with different breathing and anesthesia conditions (Table 1). Mean arterial blood pressure (MABP), pO_2_ and SaO_2_ were similar among the groups. The HR was 430‐480 bpm under isoflurane anesthesia and 200‐250 bpm under ketamine and xylazine anesthesia. The major changes of physiological parameters were pH and pCO_2_ under spontaneous breathing. With mechanical ventilation, both pH and pCO_2_ were maintained within the normal range (pH = 7.38 to 7.42, pCO_2_ = 38 to 42 mmHg^16^, ^34^) regardless of anesthesia by adjusting ventilation settings. However, mice breathing naturally under ketamine and xylazine anesthesia had low pH and high pCO_2_ levels, indicating a severe hypercapnic condition.

**TABLE 1:** Physiological parameters of three mouse groups

Group	HR (beat/min)	MABP (mmHg)	pH	PCO_2_ (mmHg)	PO_2_ (mmHg)	SaO_2_ (%)
**KX‐SB**	221.5 ± 10.50	72.9 ± 2.83	7.15 ± 0.02	83.22 ± 3.52	164.63 ± 13.37	94.11 ± 1.44
**KX‐MV**	246.3 ± 11.54	67.1 ± 1.34	7.38 ± 0.02^§^	40.56 ± 2.08^§^	155.20 ± 6.73	98.15 ± 0.25
**Iso‐MV**	448.4 ± 21.17^§^	71.0 ± 3.45	7.41 ± 0.02^§^	37.35 ± 2.04^§^	172.67 ± 2.03	98.75 ± 0.04

Abbreviations: HR, heart rate; MABP, mean arterial blood pressure; PCO_2_, partial arterial pressure of CO_2_; PO_2_, partial arterial pressure of O_2_; SaO_2_, arterial oxygen saturation level. Mean ± SEM, *n* = 8 (ketamine/xylazine with spontaneous breathing, KX‐SB), 10 (ketamine/xylazine with mechanical ventilation, KX‐MV) and 6 (isoflurane with mechanical ventilation, Iso‐MV).

ANOVA test (with Bonferroni correction) was performed among the three groups.

§ *P* < .001 compared with the KX‐SB condition.

### 3.2 Baseline vessel diameter in three different conditions

**FIGURE 1:**
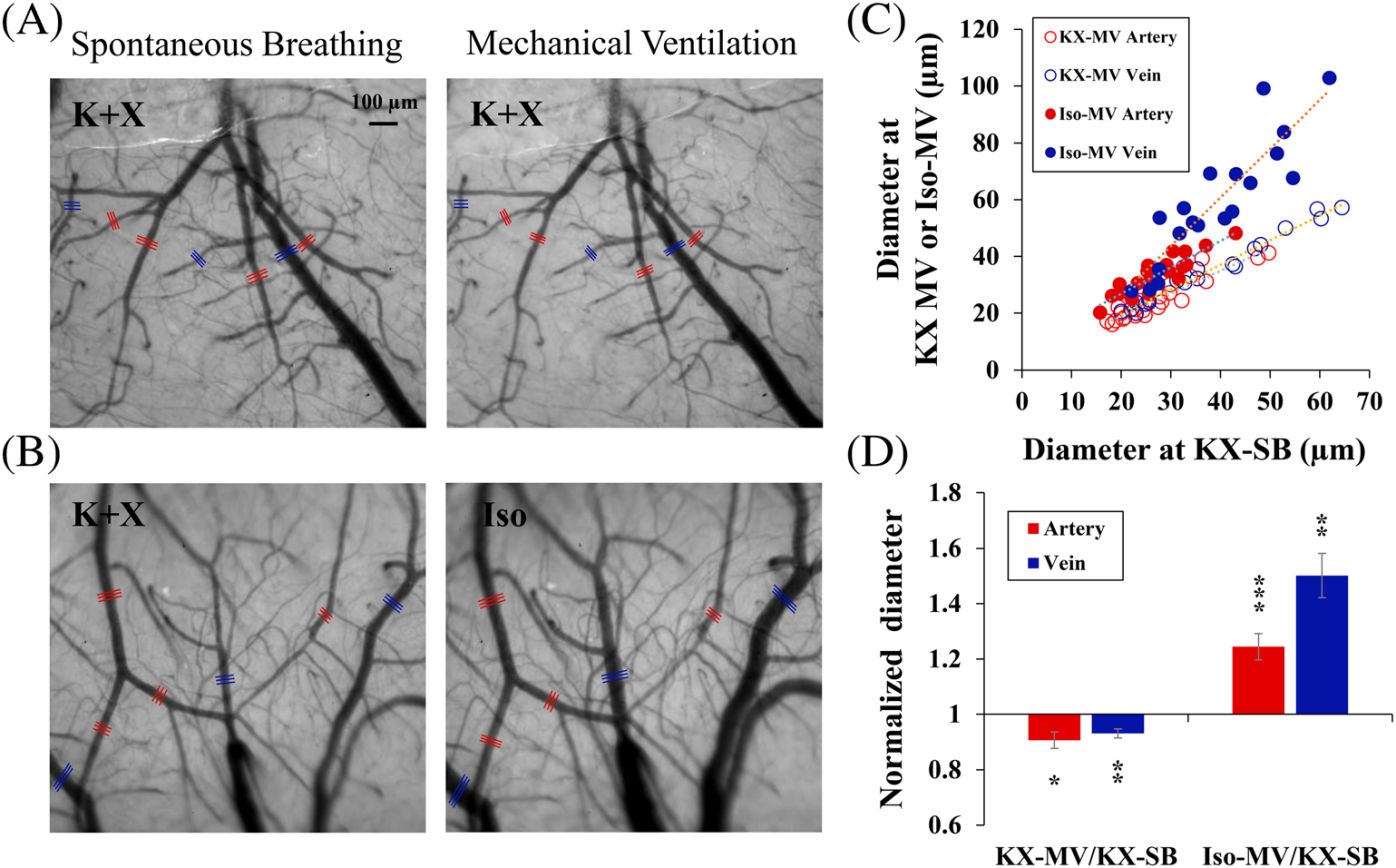
Baseline vessel diameter in spontaneously breathing ketamine/xylazine‐anesthetized mice (KX‐SB) and mechanically ventilated ketamine/xylazine (KX‐MV) or isoflurane‐anesthetized (Iso‐MV) mice. (A, B) Optical images of exposed somatosensory cortexes under a 572 nm filter in two representative mice. One mouse was used for spontaneous breathing and mechanical ventilation under ketamine/xylazine (A), and the other was used for the KX‐SB and Iso‐MV conditions (B). Red and blue lines: arteries and veins for mean diameter analysis. (C) Relationship of arterial (red) and venous vessel diameters (blue) between the KX‐SB and mechanically ventilated conditions (KX‐MV and Iso‐MV) (total 83 vessels in 12 mice). All animals were studied in the KX‐SB condition, and six mice each were subsequently used for the KX‐MV (open symbols) and Iso‐MV (filled symbols) conditions. Typically, isoflurane dilates vessels more than ketamine/xylazine. (D) Normalized vessel diameter with mechanical ventilation. Averaged baseline vessel diameters of KX‐MV and Iso‐MV were normalized with those of KX‐SB. Error bars: SEM; * *P* < .05; ** *P* < .01; *** *P* < .001

Pial vascular morphology was visualized with CBVw 572‐nm OIS in the same animals under two different conditions (Figure 1A,B). Because oxy‐ and deoxy‐hemoglobin absorb 572‐nm light equally and reduce reflected light intensity, dark lines indicate pial vessels. Because the ketamine/xylazine self‐breathing condition was used for all animals, it was considered as the reference for comparisons. When pCO_2_ was reduced to a normal level by mechanical ventilation under ketamine and xylazine anesthesia, the diameters of the pial vessels were reduced as expected (Figure 1A). However, when isoflurane was used, the vessels were dilated compared with the ketamine/xylazine condition even when the pCO_2_ was normal (Figure 1B). To compare the resting vascular diameters quantitatively, three to four arterial and venous vessels were chosen in each animal (red and blue lines in Figure 1A,B). Then their diameters were obtained under the two conditions and plotted for all animals (Figure 1C). The slopes were 0.80 (*r* = 0.94; *P* < .001; *n* = 23) and 0.87 (*r* = 0.99; *P* < .001; *n* = 20) for arteries and veins in the KX‐MV condition, respectively, and 0.93 (*r* = 0.90; *P* < .001; *n* = 21) and 1.72 (*r* = 0.95; *P* < .001; *n* = 20) for arteries and veins in the Iso‐MV condition, respectively. The most notable change was a large dilation of venous vessels when isoflurane was used. Compared with the spontaneous breathing condition under ketamine and xylazine anesthesia, arterial and venous vessels slightly constricted similarly during the mechanical ventilated condition under ketamine and xylazine anesthesia (0.91 and 0.93, respectively) and dilated largely under isoflurane anesthesia (1.24 and 1.50, respectively) (Figure 1D).

### 3.3 Hemodynamic responses evoked by 4‐second forepaw stimulation in three different conditions

**FIGURE 2:**
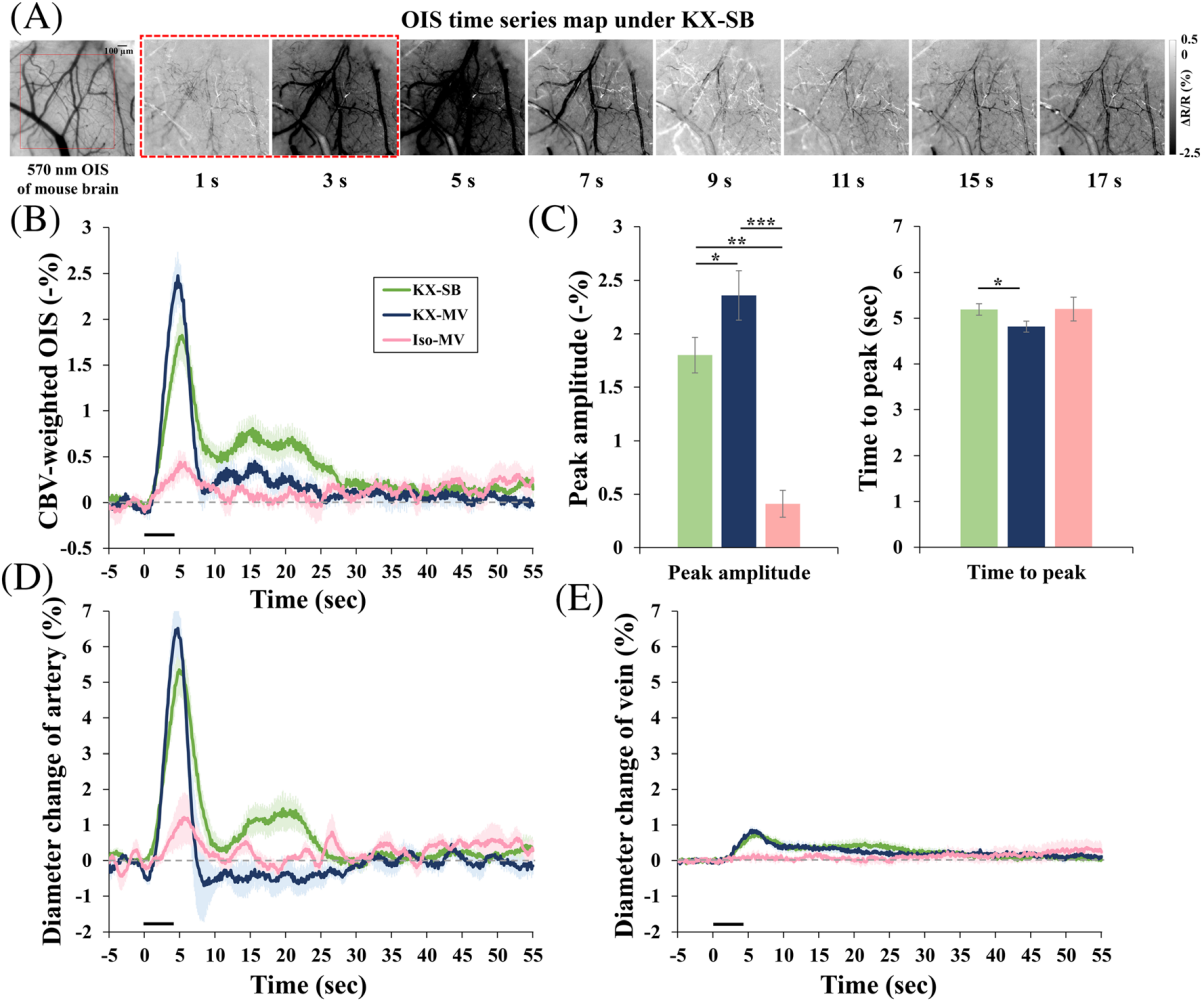
CBV‐weighted (CBVw) optical intrinsic signal (OIS) responses to 4‐second somatosensory stimulation of ketamine/xylazine‐anesthetized mice in the spontaneous breathing (KX‐SB, *n* = 12) vs. mechanical ventilation condition (KX‐MV, *n* = 6 and Iso‐MV, n = 6). (A) Reflected optical image of somatosensory cortex forepaw region with 572 nm filter (left) and functional activation maps (right) in a representative mouse under spontaneous breathing. Red square (left): ROI for time course analysis; red dashed square (right): stimulation period; ΔR/R: normalized reflectance change. Negative change indicates an increase in total hemoglobin content. (B) Averaged time course of CBVw responses in the spontaneous breathing under KX anesthesia (green), mechanical ventilation under KX anesthesia (blue) and mechanical ventilation under Iso anesthesia (pink) conditions. (C) Averaged peak amplitude and time‐to‐peak of functional responses in the spontaneous breathing under KX anesthesia (green), mechanical ventilation under KX anesthesia (blue) and mechanical ventilation under Iso anesthesia (pink) conditions. (D, E) Averaged time courses of arterial vessels (D) and venous vessels (E) in KX‐SB (green), KX‐MV (blue) and Iso‐MV (pink) conditions. Error bars: SEM; * *P* < .05; ** *P* < .01, *** *P* < .001

Because the baseline condition can modulate evoked hemodynamic responses,^29^, ^30^, ^31^, ^35^ CBVw functional responses to 4‐second forepaw stimulation were measured in the somatosensory cortical area. Figure 2A shows representative baseline 572‐nm OIS images and CBV activation maps under the hypercapnic KX condition. Under ketamine and xylazine anesthesia, forepaw stimulation reduced image intensities (darkening in the subtraction images) due to an increase in the total hemoglobin content. To compare functional responses across different breathing conditions and anesthesia, time series were obtained from the 1 mm^2^ square ROI (Figure 2A) and plotted in Figure 2B. Under ketamine and xylazine anesthesia, the hypercapnic spontaneous breathing condition induced a smaller and slower response than the normocapnic mechanical ventilation condition. A very weak response was observed under isoflurane. The CBV‐weighted peak amplitude was 1.80% ± 0.17% (*n* = 12), 2.36% ± 0.23% (*n* = 6) and 0.41% ± 0.13% (n = 6) in the KX‐SB, KX‐MV and Iso‐MV conditions, respectively (Figure 2C). Hemodynamic responses (Figure **2**C) in the KX‐MV condition (4.82 ± 0.12 seconds) were faster than those in the KX‐SB (5.19 ± 0.08 seconds) and Iso‐MV conditions (5.20 ± 0.26 seconds). Arterial and venous vessel responses to 4‐second forepaw stimulation were separated for comparisons (Figure 2D,E). As expected, arterial vessels dilated much larger and faster than venous vessels. Interestingly, the poststimulus response was observed in CBVw OIS and arterial dilation under the KX‐SB condition.

### 3.4 Hemodynamic responses evoked by 20‐second forepaw and drug stimulation in the normocapnic and hypercapnic KX conditions

**FIGURE 3:**
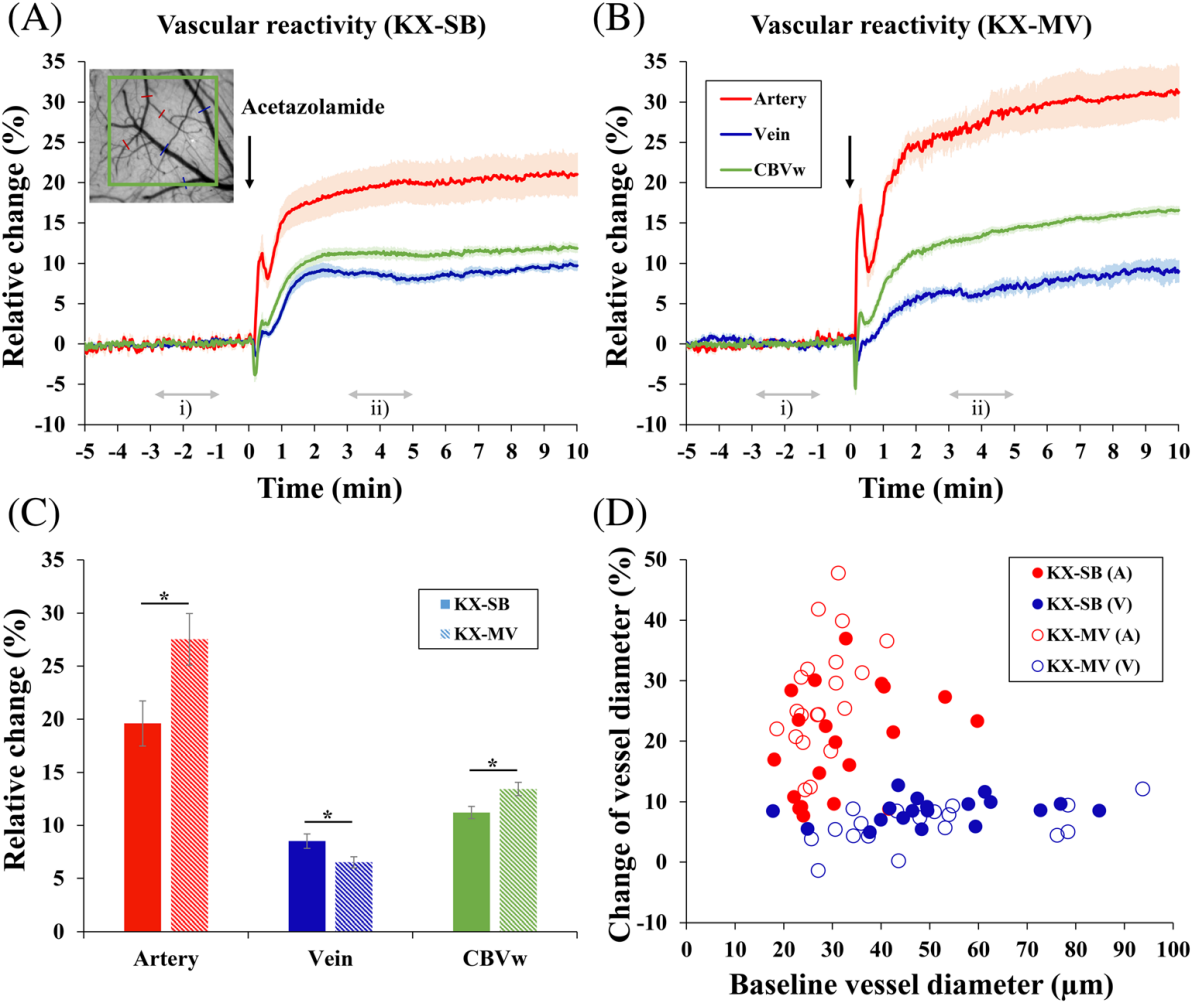
Evoked vascular responses to acetazolamide in spontaneously breathing (SB) and mechanical ventilated (MV) ketamine/xylazine‐anesthetized mice. (A, B) Averaged time courses of arterial diameter (red), venous diameter (blue) and CBVw responses (green) during the injection of the vasodilator acetazolamide (A: *n* = 6 and B: *n* = 5 animals). Inset: 572‐nm OIS; CBVw OIS was obtained from the green square ROI; arterial and venous responses from the red and blue lines, respectively. The polarity of the CBVw OIS was inverted to reflect actual CBV increases. The amplitude at the steady‐state condition was determined for the reactivity test (baseline: 2‐minute time period (i) in A and B, and stimulation: 2‐minute time period (ii)). (C) Average amplitude obtained from the 3‐5 minute period (see time period (ii) in A and B). Error bars: SEM; filled bars: spontaneously breathing ketamine/xylazine; right hatched bars: mechanically ventilated ketamine/xylazine; * *P* < .05 (D) Relative diameter changes in individual vessels (as a function of baseline diameter) induced by the injection of the vasodilator acetazolamide (39 vessels in KX‐SB and 38 vessels in KX‐MV)

Under the severe hypercapnia induced by the KX‐SB condition, vascular reactivity is likely to be hampered. Therefore, we further examined arterial and venous responses by acetazolamide stimulation under different breathing conditions under ketamine and xylazine anesthesia (Figure 3). Right after the injection (indicated by a downward arrow in Figure 3A,B), an initial decrease and overshoot were observed, which is likely due to the side effects of a bolus injection. As expected, the vessel reactivity of arteries and CBV in the normocapnic condition (KX‐MV) was greater than that in the hypercapnic condition (KX‐SB). Veins responded similarly in both breathing conditions. The average reactive response of the artery, CBVw OIS and vein was almost 20%, 12% and 9% in the KX‐SB condition and 28%, 15% and 7% in the KX‐MV condition, respectively (Figure 3C). Similar to the functional response, arteries responded faster and larger than veins (Figure 3A‐D). To determine vessel‐size dependent responses, the changes averaged over the 3‐5 minute postinjection period were plotted as a function of baseline vessel diameter (Figure 3D). Arterial vessel dilation (red symbols) is related to baseline diameter, while venous vessels (blue symbols) are independent of vessel size.

**FIGURE 4:**
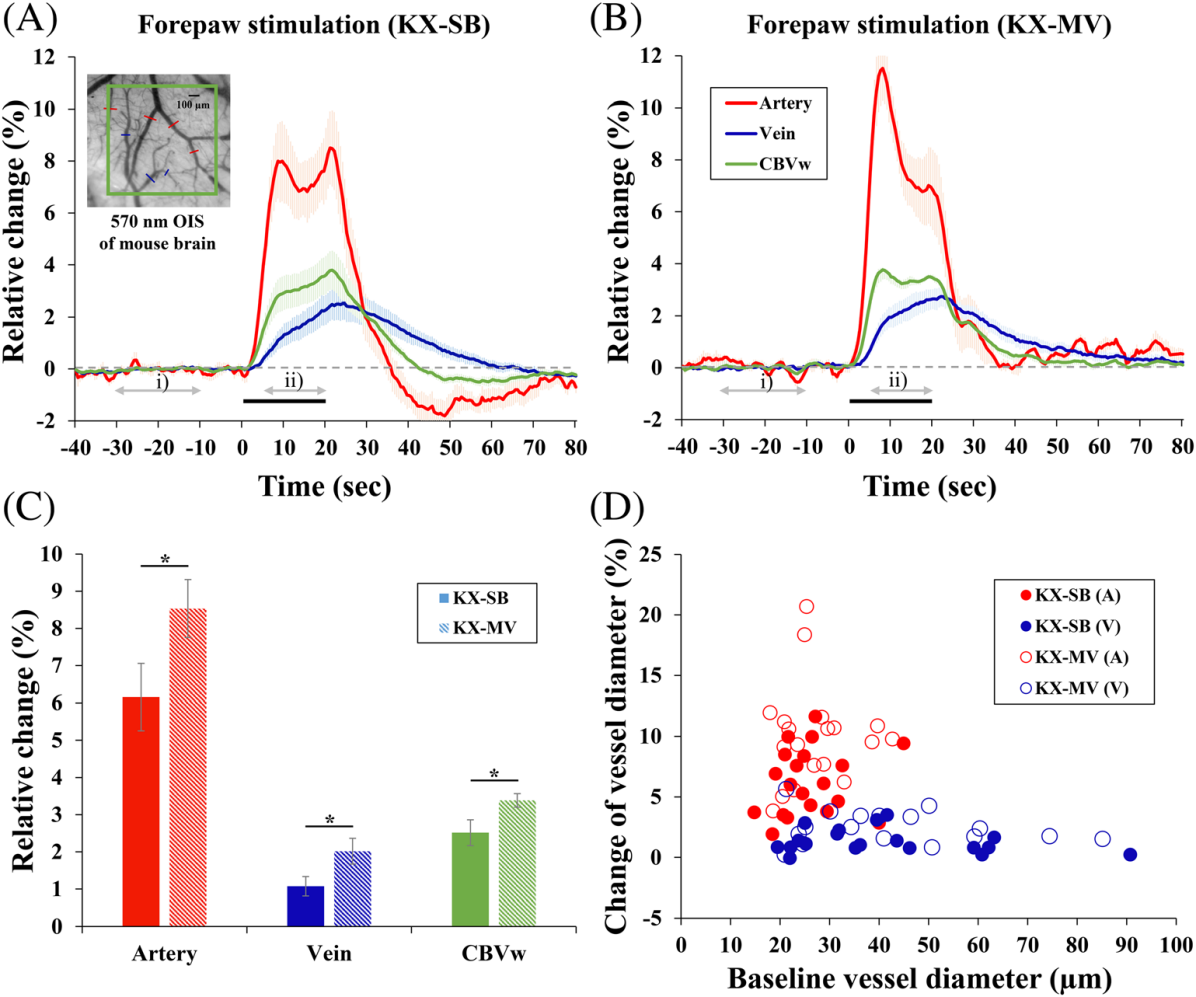
Evoked vascular responses to 20‐second forepaw stimulation in spontaneously breathing (KX) and mechanical ventilated(MV) ketamine/xylazine‐anesthetized mice. (A, B) Averaged time courses of arterial diameter (red), venous diameter (blue) and CBVw responses (green) during 20‐second forepaw stimulation (A: *n* = 6 and B: *n* = 5 animals). The amplitude was determined as an average of 5‐20 seconds of data (marked ii) period) after the onset of stimulation for forepaw stimulation. (C) Average amplitudes of arterial dilation (red), venous dilation (blue) and CBVw OIS (green). Error bars: SEM; filled bars: spontaneously breathing ketamine/xylazine; right hatched bars: mechanically ventilated ketamine/xylazine; * *P* < .05. (D) Relative diameter changes in individual vessels induced by forepaw stimulation (42 vessels in KX‐SB and 37 vessels in KX‐MV)

To compare hemodynamic responses with the fMRI studies, 20‐second forepaw stimulation was used to examine arterial and venous dilation, along with CBVw OIS in spontaneously breathing or mechanically ventilated KX‐anesthetized mice (Figure 4). Commonly, arterial vessels dilated quickly and peaked within 10 seconds of the onset of stimulation, whereas venous vessels dilated slowly, peaking after the end of the 20‐second stimulation period (Figure 4A,B). Since the CBV response from the square ROI shows a time course pattern that is a mixture of arterial and venous responses (Figure 4A,B), the CBV response also reflects arterial changes. Average response of vessel diameters and CBV‐weighted OIS was larger in KX‐MV compared with the KX‐SB condition (Figure 4C). Interesting observations were (i) two peaks of arterial responses during the stimulation period and poststimulus undershoots of arterial responses in the KX‐SB condition (Figure 4A), and (ii) large overshoots of arterial responses in the KX‐MV condition (Figure 4B). The magnitude of poststimulus undershoots (an average of 40 to 60 seconds) was −1.37% ± 0.69% and − 0.27% ± 0.09% in artery and CBV response, respectively. To determine vessel size‐dependent responses, the change averaged over the 5‐20 seconds stimulation period was plotted as a function of baseline vessel diameter (Figure 4D). The magnitude of arterial dilation ranged from 2% to 21% (red symbols), whereas venous responses were less than 6% (blue symbols), irrespective of baseline vessel size. The vessel‐size dependent responses induced by forepaw stimulation (Figure 4D) have similar properties to those induced by vasodilator (Figure 3D).

### 3.5 BOLD fMRI of 20‐second forepaw stimulation in the normocapnic and hypercapnic KX conditions

**FIGURE 5:**
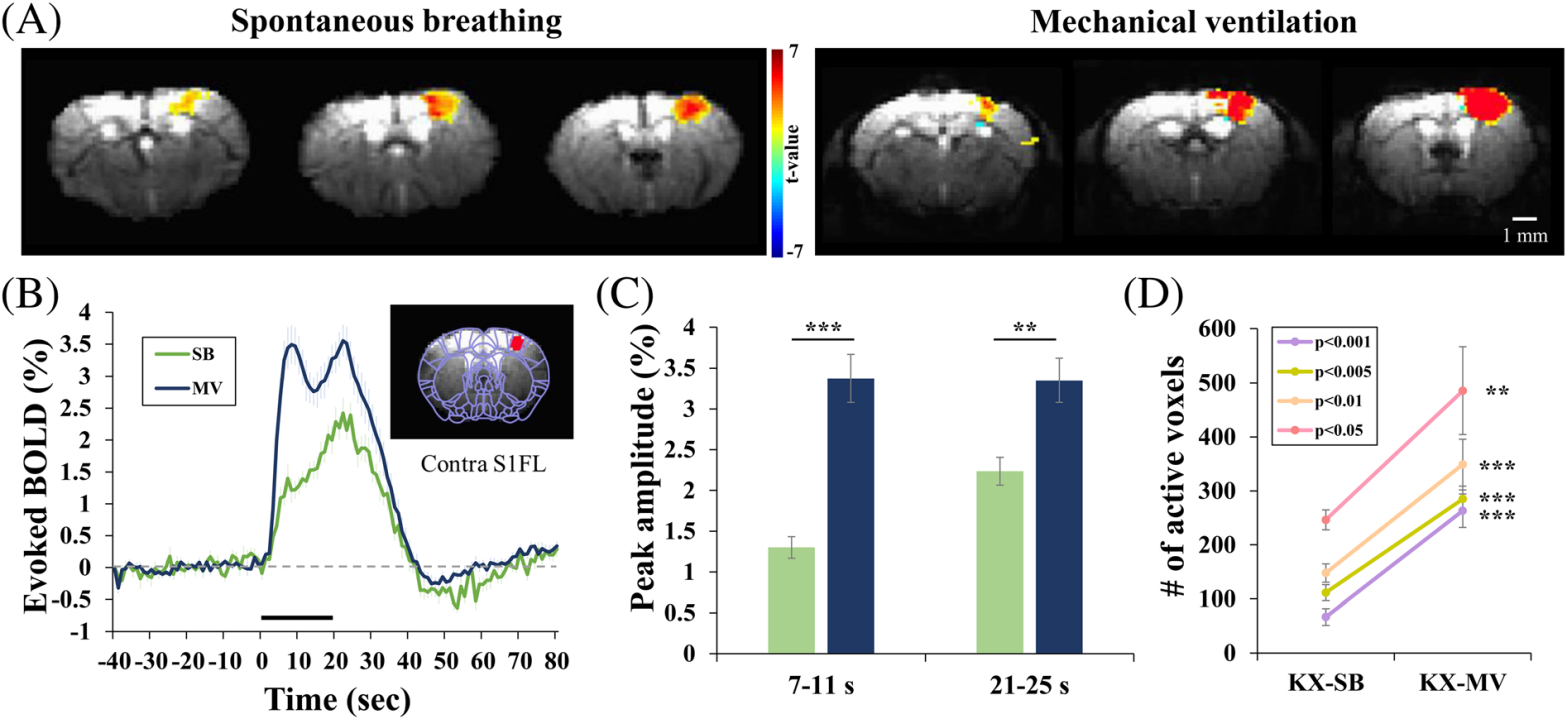
BOLD fMRI responses to 20‐second forepaw stimulation under spontaneous breathing (SB) or mechanical ventilation (MV) in ketamine/xylazine‐anesthetized mice. (A) Activation maps of two representative mice overlaid on the original EPI images. Localized activation was observed at the contralateral forelimb area. (B) Averaged time courses from the contralateral primary somatosensory (S1FL) cortical ROI (red sphere ROI in inset figure). The mechanical ventilation condition induced a larger and faster response. (C) Average amplitude obtained from the 7‐11 sec and 21‐25 second periods. (D) The number of active voxels in the contralateral S1 regions at four different thresholds; *P* < .05, *P* < .01, *P* < .005 and *P* < .001. Error bars: SEM; ** *P* < .01; *** *P* < .001

For the fMRI studies, the same 20‐second stimulation protocols used in the OIS studies were performed on spontaneously breathing or mechanically ventilated KX‐anesthetized mice. Figure 5A shows the GLM‐based activation maps from the 20‐second forepaw stimulation of representative mice. In both hypercapnic and normocapnic conditions, well‐localized BOLD activity was detected in the contralateral S1FL region, with higher statistical values under mechanical ventilation. No significant activity was observed at the ipsilateral S1FL, but small clusters were often identified in the secondary somatosensory (S2) region in the mechanically ventilated mice. To compare evoked BOLD response changes, a time series was obtained from the sphere ROI (inset figure in Figure 5B). Similar to the OIS results, smaller and slower BOLD signal changes were observed under spontaneous breathing than mechanical ventilation (Figure 5B,C). Unlike the normocapnic mechanical ventilation condition, the BOLD response in the hypercapnic spontaneous breathing condition increased slowly from the initial hump at ~9 seconds to the major peak at ~23 seconds (Figure 5B), which is similar to the observation in arterial dilation (Figure 4A). Poststimulus BOLD undershoot (Figure 5B) was observed in the spontaneous breathing condition, which is also similar to OIS data (Figure **4**A). The averaged BOLD changes for spontaneous breathing (*n* = 11) and mechanical ventilation (*n* = 11) were 1.30% ± 0.13% and 3.37% ± 0.29%, respectively, for the 7‐11 second period and 2.24% ± 0.17% and 3.35% ± 0.27%, respectively, for the 21‐25 second peak period. Consequently, the active voxel sizes were larger in all thresholds (*P* < .05, *P* < .01, *P* < .005 and *P* < .001) in the mechanical ventilation condition (263 ± 30, 285 ± 24, 349 ± 47 and 485 ± 81 voxels) than in the spontaneous breathing condition (67 ± 16, 111 ± 14, 148 ± 17 and 246 ± 18 voxels) (Figure 5D).

## 4 DISCUSSION

We have successfully characterized the baseline and evoked hemodynamics responses in two anesthetics. Under 1.0%‐1.1% isoflurane anesthesia, normal physiological conditions were maintained by mechanical ventilation, but baseline vessels significantly dilate, and forepaw stimulation‐induced responses are very small. Under ketamine and xylazine anesthesia, spontaneous breathing and mechanical ventilation conditions were compared. Our major findings are: (i) spontaneous breathing induces severe acidosis and hypercapnia, but it dilates the baseline arterial and venous vessels only slightly compared with the normocapnic mechanical ventilation; and (ii) the evoked response to forepaw and acetazolamide stimulation is slower and smaller in the hypercapnic condition than in the normocapnic condition. The most surprising finding is that even though spontaneous breathing induced severe hypercapnia, significant evoked functional responses were observed in CBVw optical imaging, vessel diameter and BOLD fMRI under ketamine and xylazine anesthesia.

### 4.1 Baseline vascular physiology under anesthesia

Anesthesia alters the mechanics of the respiratory system and gas exchange in the lungs, reducing respiratory functions.^19^, ^28^, ^36^, ^37^, ^38^, ^39^, ^40^, ^41^ Under spontaneous breathing, the blood oxygen saturation level decreases, which can be remedied by increasing the inspired oxygen fraction to ~0.3. Anesthesia also impairs CO_2_ elimination from the lungs, which can be addressed by using proper mechanical ventilation. Thus, mechanical ventilation with intubation or tracheotomy is performed in anesthetized studies to maintain normal blood gas levels. We carefully adjusted our ventilator settings based on arterial blood gas analyses to achieve normal vascular physiology. Under the normal blood gas condition, arterial and venous vessel diameters are ~ 35% and 55% larger under 1.0%‐1.1% isoflurane than under ketamine and xylazine anesthesia. Arterial and venous CBV under isoflurane are expected to increase to ~82% (eg 1.35^2^–1) and 140% (1.55^2^–1), respectively, compared with ketamine and xylazine anesthesia. This indicates that the baseline CBV can be >100% higher under isoflurane due to the vasodilatory property of isoflurane anesthetics.^37^ The forepaw‐induced response under isoflurane is small, unlike many rat fMRI studies, in which the evoked response is reliably obtained.^20^, ^38^ The exact cause of differences between two species is unknown, but we speculate that mouse has high susceptibility to isoflurane, also dependent on the strain (in our case, the commonly used C57BL/6 strain).^25^, ^42^

Ketamine and xylazine anesthesia reduced HR (~220 bpm) and pH (~7.15) and increased pCO_2_ (~80 mmHg) under spontaneous breathing. Arras et al^28^ reported HRs of ~220 bpm (530 bpm awake), pH of ~7.2 (7.45 awake) and pCO_2_ of ~70 mmHg (~25 mmHg awake) when 150 mg/kg ketamine and 30 mg/kg xylazine were used (Protocol 2 in Arras et al^28^). Small differences between our results and those in Arras et al^28^ are likely caused by the difference in the ketamine and xylazine dose. Elevated pCO_2_ causes astrocytes to release prostaglandin E2, a vasodilator,^43^ and acidosis increases nitric oxide release, which will relax cerebral vascular smooth muscle.^10^, ^44^ Interestingly, severe hypercapnia induces the dilation of pial vessels by only ~10% compared with the normocapnic condition under ketamine and xylazine anesthesia. Assuming the finding for the pial vessels can be translated to the parenchymal vessels, then baseline CBV is increased by ~20% when pCO_2_ increases from 40 to ~80 mmHg. Grubb et al^10^ determined the relationship between pCO_2_ and CBV in anesthetized rhesus monkeys and found that CBV (ml/100 g) = 0.041 × pCO_2_ (mmHg) + 2. If we adapt that relation to our mouse data, the baseline CBV is 3.6 and 5.3 ml/100 g at 40 and 80 mmHg pCO_2_, respectively. The expected CBV increase with elevated pCO_2_ is ~50%, which is much larger than in our experimental data. The exact cause of the difference is not known, but it is likely due to the properties of the different anesthetics used (phencyclidine vs. ketamine and xylazine).

### 4.2 Evoked responses to stimulation under ketamine and xylazine anesthesia

Changing the baseline pCO_2_ level affects the evoked response. In awake humans, the relationship between baseline conditions and evoked BOLD or CBF responses has been investigated.^8^, ^29^, ^45^, ^46^, ^47^, ^48^, ^49^, ^50^ Some studies reported that the evoked changes remained constant irrespective of the baseline condition,^45^, ^49^, ^51^ whereas others reported that the evoked response was reduced by hypercapnia.^29^, ^50^, ^52^ These inconsistent observations could be due to different experimental designs and methods. However, an increase in the time‐to‐peak of the evoked responses with an increase in the baseline pCO_2_ level appears to be consistent.^8^, ^29^

In anesthetized rat studies using isoflurane^31^, ^35^ and urethane,^30^, ^53^ somatosensory stimulation‐induced hemodynamic responses decreased significantly with severe hypercapnia. The BOLD response to forepaw stimulation under 1.1%‐1.3% isoflurane was similar at a PCO_2_ of 49 mmHg^32^ and slightly reduced at a PCO_2_ of 54 mmHg^36^ compared with the normocapnic condition, but it was almost eliminated at a PCO_2_ of 70 mmHg.^31^ Similarly, Jones et al^30^ reported no significant difference in the hemodynamic changes caused by a 3‐second whisker stimulation at a PCO_2_ of 48 mmHg compared with the normocapnic condition and a significant reduction in the responses at a PCO_2_ of 75 mmHg under urethane. To evaluate the dynamics of the CBV responses, 16‐second whisker stimulation was used in the normo‐ (31 mmHg) and hypercapnic conditions (76 mmHg) under urethane.^53^ In the normocapnic condition, the CBV‐weighted OIS response peaked at 3.1 seconds after the onset of stimulation and remained at a plateau during the stimulation period, whereas during hypercapnia, no initial peak was detected, and the hemodynamic response slowly increased to the peak at the end of stimulation. In previous anesthetized mouse fMRI experiments that did not measure baseline blood gases, the time‐to‐peak response to a 20‐second stimulation was 18‐22 seconds.^4^, ^7^, ^24^, ^25^ This slow time‐to‐peak is likely caused by baseline hypercapnic status.

Our mouse data with ketamine and xylazine anesthesia show that the hemodynamic peak magnitude decreased by 25%‐33% with hypercapnia (~80 mmHg) and 4‐20 seconds stimulation, which is somewhat similar to previous findings in anesthetized rats.^30^, ^31^, ^53^ At the normocapnic condition during 20‐second stimulation, CBVw OIS and BOLD response evoked by forepaw stimulation peaked at ~ 9 seconds, then decreased slightly and peaked again at the end of stimulation (Figures 4B and 5B).^24^ This two‐peak effect was not observed in rat forepaw stimulation studies under various anesthetics,^18^, ^19^, ^20^, ^21^ and in mouse forepaw stimulation studies under different anesthesia.^7^, ^25^ Secondary OIS and BOLD peak observed under the normocapnic condition (Figure 4B green trace and Figure 5B blue trace) disappeared under the hypercapnic condition. Similarly, the poststimulus undershoots of CBVw OIS and BOLD responses under the hypercapnic condition (Figures 4A and 5B, green traces) disappeared under the normocapnic condition. These observations indicate that the secondary peak and poststimulus undershoot are closely associated with vascular physiology under ketamine and xylazine anesthesia.

At the hypercapnic spontaneous breathing condition, the response had a hump at ~9 seconds and then increased slowly and continuously to the peak at the end of stimulation. The slower and smaller response at higher pCO_2_, which agrees with data observed in humans, is likely caused by intrinsic vascular properties.^29^ However, we cannot exclude the possibility that the evoked neural activity is also reduced by the hypercapnia and that neurovascular coupling is intact in both conditions.^53^ Further studies are needed to measure the neural activity in both the normo‐ and hypercapnic conditions.

We investigated dynamic changes in the arterial and venous vessels with 20‐second forepaw stimulation and vasodilatory drug injection. The arterial vessels respond faster and larger than the venous vessels. During the 20‐second stimulation (Figure 3A), the arterial vessels dilated quickly, peaking within 10 seconds of stimulation onset, whereas the venous vessels dilated slowly until the end of stimulation. It is well‐accepted that arterial dilation is faster and larger than venous dilation. The magnitude and dynamics of venous dilation depend on the species, anesthetics and stimulation type and duration.^19^, ^54^, ^55^, ^56^, ^57^, ^58^ Previous forepaw stimulation studies found no significant venous volume changes with 15 to 20 seconds stimuli in isoflurane‐anesthetized rats^55^, ^56^ and rapid venous (though still slightly slower than arterial) dilation with 10‐second stimuli in dexmedetomidine‐anesthetized rats (Figure 9C in Fukuda et al^19^). Our arterial and venous time constants of 5.8 (5.6) and 16.3 (14.8) seconds in the hypercapnic (normocapnic) condition, respectively, are somewhat similar to findings of arterial and venous time constants of 8 and 44 seconds, respectively, in 30‐second whisker stimulation studies of awake mice,^57^ and 2.9 and 13.5 seconds, respectively, in 40‐second forepaw stimulation of α‐chloralose‐anesthetized rats.^58^

Although the magnitude of arterial and venous dilation depends on the proximity to active sites and the vessel size, arterial dilation is 2‐4 times larger than venous dilation.^57^, ^59^ Similar findings were observed in our vascular reactivity test of acetazolamide. Generally, it is expected that smaller vessels have larger percentage changes.^60^ But in our acetazolamide and forepaw stimulation studies of ketamine and xylazine‐anesthetized mice, the larger arterial vessels dilated more, whereas the venous vessel dilation was independent of diameter. This observation could be due to our small sample size.

### 4.3 Mechanical ventilation vs. spontaneous breathing

To maintain normal physiological conditions under anesthesia, it is critical to use mechanical ventilation. We used an endotracheal intubation procedure. However, our intubated mice did not resume the normal bodyweight gain of 1‐2 g per week after they recovered, so they were not used for repeated experiments. This result was likely due to the side effects of intubation, such as sore throat and pain. To perform longitudinal fMRI studies, improved intubation skills are essential. To achieve a normal physiological condition, tracheotomy or intubation is essential, and blood gas sampling is also needed. In many previous fMRI studies, intubation was performed without blood gas analyses.^5^, ^6^, ^61^ In those cases, controlling an animal's physiological condition often relies on end‐tidal CO_2_, which should be calibrated with a blood gas analysis. In our case, arterial blood gas data were used to determine the ventilator settings, which were then adopted in our later optical and fMRI studies. The disadvantages of mechanical ventilation are its invasiveness. In addition to intubation, arterial catheterization in mice is technically demanding, and the potential for repeated sampling of arterial blood is limited by the small blood volume. The major advantage of mechanical ventilation is maintaining a normal physiological range and maximizing functional responses, which is beneficial for comparisons across different animal groups.

Spontaneous breathing is simple, easy to implement, and does not require any intubation or arterial catherization, which eliminates the risk of complications (suffocation, bleeding, pain, etc.). The anesthetized, spontaneously breathing mice successfully recovered from anesthesia and quickly regained their normal weight, permitting us to perform other fMRI experiments with them. The disadvantages of spontaneous breathing are hypercapnia and acidosis caused by the impaired gas exchange, which lead to sluggish and submaximal functional responses.^28^ The most important advantage is the ability to conduct longitudinal fMRI studies over time.^24^

## 5 CONCLUSION

We used OIS and BOLD fMRI to characterize the baseline and evoked responses of mice under spontaneous breathing and mechanical ventilation with ketamine and xylazine anesthesia. The spontaneous breathing condition induced severe hypercapnia at baseline, but significant vascular reactivity and evoked functional responses were obtained. Mechanical ventilation allowed us to maintain the mice in a normal physiological condition, which induced larger and faster hemodynamic responses. These results suggest that even though mechanical ventilation provides higher functional responses, spontaneous breathing is still viable for mouse fMRI studies under ketamine and xylazine anesthesia due to its simplicity.

## FUNDING INFORMATION

This project was funded by the Institute for Basic Science (IBS) in Korea (IBS‐R015‐D1).
